# Cavernous Hemangioma in the Orbital Cavity: Case Report

**DOI:** 10.1055/s-0041-1732948

**Published:** 2021-10-21

**Authors:** José Afonso de Almeida, Paula Vitória Bido Gellen, Daniel Martins Hiramatsu, Mariana Araújo dos Santos, Larissa Bitencourt, Eduardo Fagury Videira Marceliano, Michelle Paiva Weydt Galhardi, Marília F. Marceliano-Alves, Eduardo Fernandes Marques

**Affiliations:** 1 Department of Endodontics, President Antonio Carlos University - Porto Nacional – ITPAC - Porto/FAPAC, Palmas, Brazil; 2 Department of Surgery, Head, and Neck, Palmas Public General Hospital, Palmas, Brazil; 3 Dental Clinic Department, Brazilian Army General Hospital of Belem, Belem, Brazil; 4 Department of Dental Materials, Sao Jose University, Rio de Janeiro, Rio de Janeiro, Brazil; 5 Department of Endodontics, Iguaçu University, Nova Iguaçu, Brazil

**Keywords:** hemangioma, cavernous, oral pathology

## Abstract

Cavernous hemangiomas are benign malformations of vascular origin, usually well circumscribed and slow to grow. These lesions can be asymptomatic, being discovered unintentionally in imaging exams or symptomatic, indicated mainly by the presence of proptosis, diplopia, and visual disturbances by optic nerve compression. The complementary exams involve computed tomography associated with contrast, color Doppler, magnetic resonance, and angiography. Treatment can be conservative or surgical depending on the case, and the open therapy usually involves lateral, supraorbital, transconjunctival, transantral, pterional, transnasal, and extradural endoscopic orbitotomy. The present study aimed to report a recurrent case of hemangioma in the orbital cavity signaled by ocular proptosis, hyperemia, and ocular pain.The lesion was achieved through the Weber-Ferguson access with zygomatic osteotomy and preservation of the infraorbital nerve. The excision of the lesion was performed, and the previously displaced fragments were fixed with 1.5 mm mini plates. The patient has a chance of progressing with visual impairment due to considerable manipulation of the optic nerve and is being followed up.The reported case showed a successful diagnosis and therapeutic conduct, remaining now in the evolution and follow-up scenario.

## Introduction

Vascular malformations can be classified into four categories: arteriovenous malformations; cerebral cavernous malformations, also known as hemangiomas or cavernomas; venous angiomas; and finally capillary telangiectasias. Among these, hemangiomas occupy the second place with the highest incidence, second only to arteriovenous malformations. 1 However, this pathology takes the lead when treating vascular lesions in the orbital cavity. 1

It is a benign lesion with slow growth, composed of an endothelial proliferation that appear during angiogenesis with irregular sizes. 1 2 They are usually well circumscribed and have no potential for malignancy. 1

It is estimated that 80% of orbital hemangiomas are housed in the intraconal compartment (between the Tenon’s capsule and the extraocular muscles) usually in the lateral direction. 3 In addition, the prevalence encompasses women and generally occurs in middle age. 3

It is worth pointing out that they are usually asymptomatic, but patients with this tumor may present visual symptoms, such as ptosis and vision deficits by compression of the second cranial nerve pair—the optic nerve—in addition to psychological and aesthetic impairment. 1 4 5 Preoperative evaluation is essential and makes the use of imaging tests essential to carry out an adequate planning, with computed tomography (CT) and magnetic resonance imaging being the first choices. 6

Thus, the current study aimed to report a case of a hemangioma diagnosed in the orbital cavity in a 40-year-old patient.

## Case Report

**Fig. 1:**
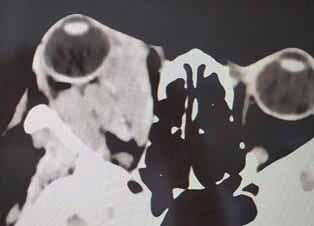
Magnetic resonance imaging showing retro-orbital and inferior-medial tumor mass.

A 40-year-old female patient presented to the head and neck surgery service with a report of protrusion of the right eyeball in early 2019. A magnetic resonance imaging (MRI) exam was requested to complement the diagnosis, showing a tumor mass positioned in a retro-orbital and inferior-medial form ( Fig. 1 ). The case was treated by excision of the tumor through infraorbital access in the direct hemi-face and remained asymptomatic for 6 months.

**Fig. 2:**
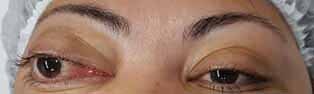
Exophthalmos, indicating tumor recurrence.

However, in September 2019, the progressive exophthalmos was observed, being indicated as a potential sign of recurrence ( Fig. 2 ). Thus, we opted for another therapeutic approach through embolization and the patient was referred to other cities in the country. However, there was no success, leading to the return to the hospital of origin.

**Fig. 3:**
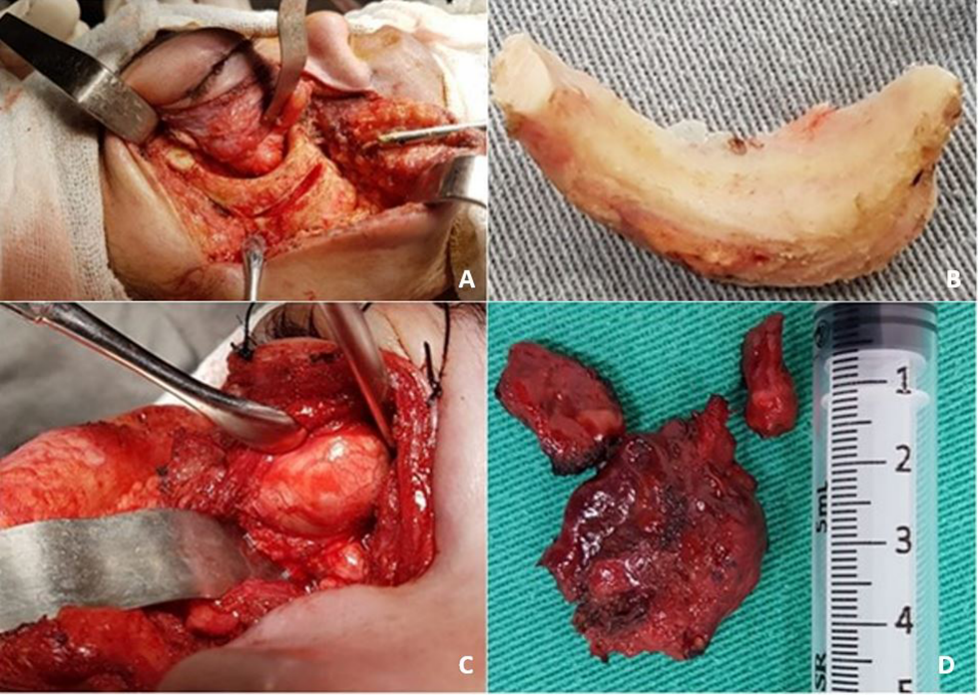
( **A** ) Weber-Ferguson access; ( **B** ) osteotomized zygomatic bone; ( **C** ) excision of the lesion; and ( **D** ) excised hemangioma.

This fact was accompanied by signs of evolution represented by ocular proptosis, hyperemia, and ocular pain of progressive character indicating the need for surgical intervention. Thus, we opted for the Weber-Ferguson approach, with tissue divulsion and exposure of the zygomatic bone ( Fig. 3A ). The visualization was enlarged through zygomatic osteotomy to provide better access to the lesion that extended from the medial part to the bottom of the orbital cone ( Fig. 3B ). In sequence, an osteotomy was performed on the frontozygomatic suture, along the contour of the maxillary anterior wall to the infraorbital ridge in the medial orbital region, with preservation of the infraorbital nerve. Finally, osteotomy followed the floor of the orbit to the frontozygomatic suture, allowing the mobilization of these fragments and providing adequate access to the tumor.

**Fig. 4:**
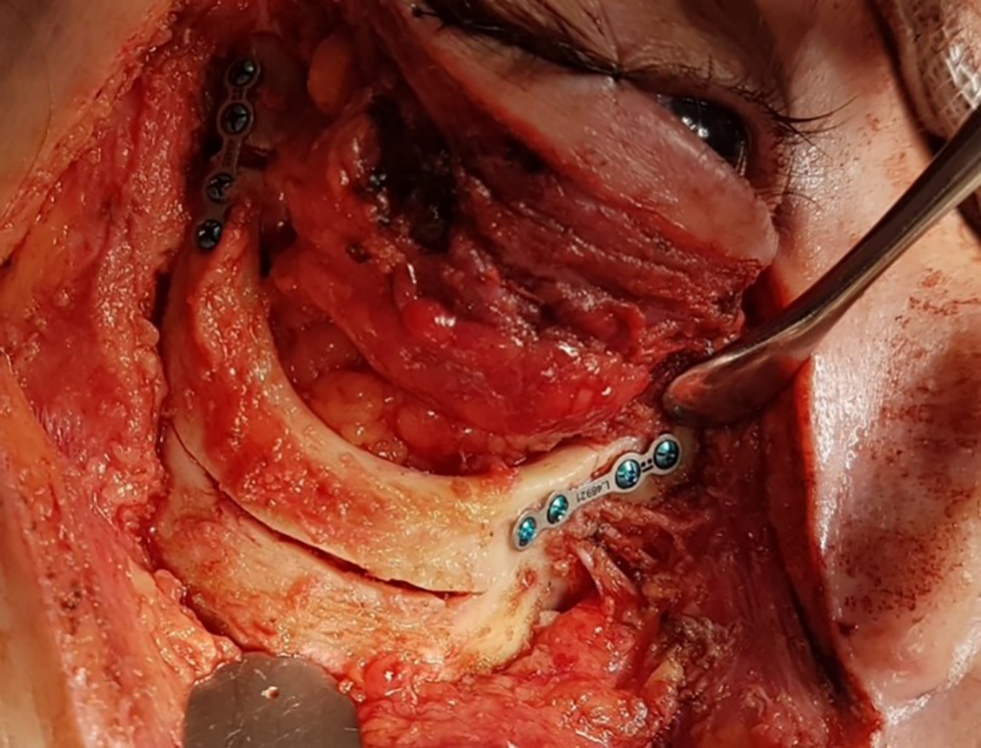
Fixation of the zygomatic and frontozygomatic suture with 1.5 mm titanium miniplates.

**Fig. 5:**
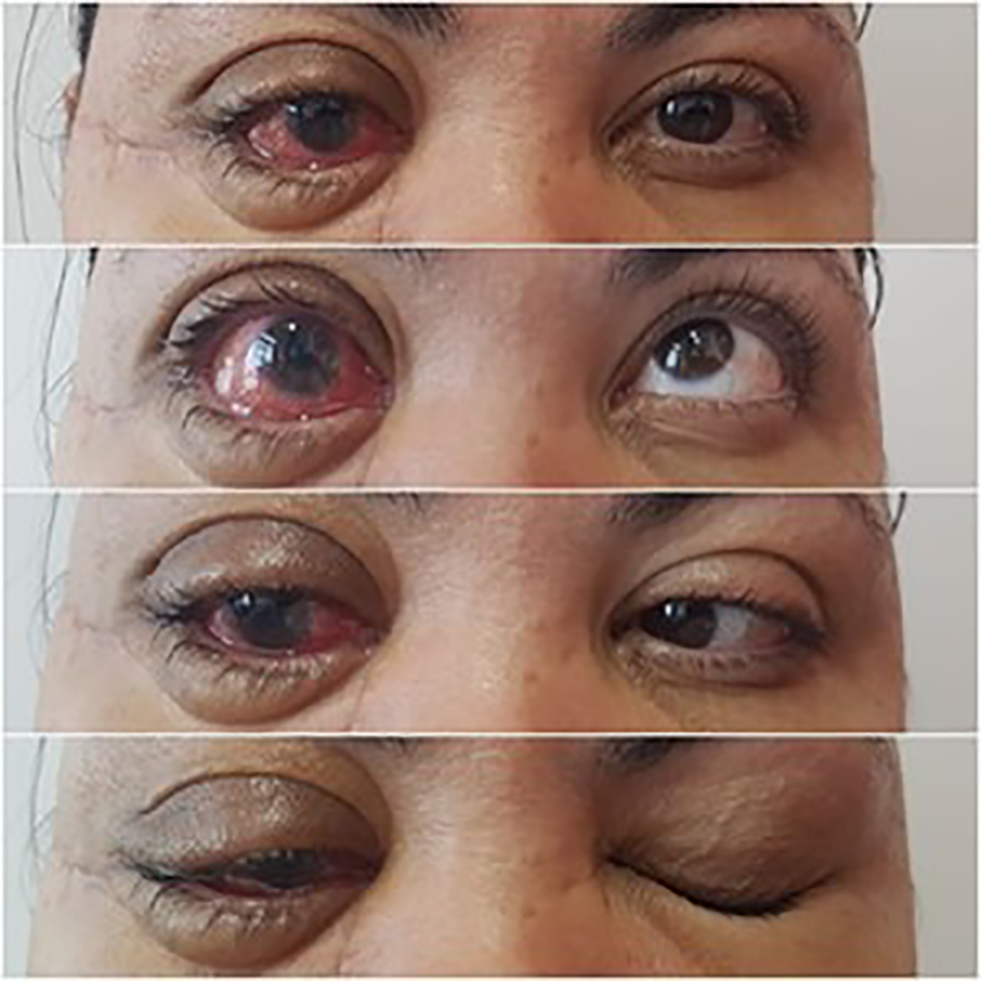
( **A–C** ) Restriction of eye movements and ( **D** ) inability to close the eye.

A complex resection was performed because it is a lesion occupying the entire posterior orbit and involvement of the optic nerve and eye movement muscles ( Fig. 4A ). After the exeresis ( Fig. 4B ), the fragments previously moved through the osteotomy were repositioned and fixed with titanium mini plates of the 1.5 mm system ( Fig. 4 ), and a drain was fixed in the lateral orbital region to be sutured ( Fig. 3C ). The patient is undergoing a 2- and 6-month, and 1- and 2-year postsurgical follow-up, with an ophthalmologist, showing signs of ocular motor restriction, light diplopia, and inability to close the eye remained ( Fig. 5A–D ).

## Discussion

Cavernous orbital hemangiomas (HCOs) are benign circumscribed vascular malformations and with a slow growth development (1–5) and may in some cases present acutely. 3 They are usually found in middle-aged adults and represent about 4 to 6% of all the intraorbital masses of the orbit/retrobulbar masses of the orbit. 1 5 7 More than 80% of orbital cavernous hemangiomas are in the intraconal compartment, with painless progressive proptosis being the most common form of presentation. 3

Das et al 2 and Calandriello et al 7 state that this lesion is more common in women, probably due to circulating estrogen/progesterone levels affecting the progression of orbicular cavernous hemangioma. In agreement with the case reported here, which refers to a female patient, as it corroborates with other studies analyzed. 1 2 8

As for the etiology of HCO, it is understood that the most common cause may result from an underlying orbital vascular anomaly: acute orbital hemorrhage from normal vessels after trauma and valsalva maneuvers in compromised patients, or from the presence of venous-lymphatic malformations, and arteriovenous malformations, in addition to neoplastic vessels (tumors). 3

Cavernous hemangiomas of the orbit can be asymptomatic, being discovered accidentally on imaging studies, or symptomatic, with the most common manifestations being proptosis, pain, diplopia, and visual disturbances due to compression of the optic nerve. 1 3 5 In the case reported in this study, the patient was symptomatic after recurrence of the lesion, with ocular proptosis, hyperemia, and progressive eye pain. Symptoms are usually reversible unless the lesion changes the axial length or optic nerve function, thereby causing irreversible visual impairment. 7

To confirm possible diagnostic hypotheses, it is interesting that the surgeon uses complementary exams, with CT, associated with the use of contrast dye, the most common method. 2 5 8 However, magnetic resonance imaging (MRI), color Doppler examinations, and angiography are also methods used to identify the location and extent of the hemangioma. 5 In this scenario, in agreement with some case reports, 3 4 5 the patient in this study underwent an MRI scan, which identified the location of the lesion in the retro-orbital and inferior-medial region.

These exams can assist in the proper establishment of the pathology, thus ruling out other diseases of orbital cavernous malformations. Some types of cysts, vascular and peripheral nerve injuries, tumors of the optic and meningeal nerve, and capillary hemangioma or lymphangioma can be considered differential diagnoses, which must be carefully analyzed to establish an appropriate therapeutic approach. 5

It is noteworthy that HCOs are generally identified because they are caves lined with blood-filled endothelium, which differentiate them from other orbital vascular tumors, such as capillary hemangiomas, hemangiopericytomas, hemangioendotheliomas, and angiofibromas. 1

After the diagnosis is established, the surgeon must plan the patient’s treatment plan according to the peculiarities of the case. Treatment may be surgical or not, and this will depend on the location and complexity of the lesion. The most common surgical approaches are lateral, supraorbital, transconjunctival, transantral, pterional, transnasal, and extradural endoscopy. 1 5 7

The HCOs located in the medial orbital compartment, lower, and at the orbital apex, are indicated for transnasal endoscopic approach. This approach generates orbital decompression, even in cases with partial removal of the lesion. 7

Lenzi et al 9 and Bleir et al 10 reported in their studies the use of the transnasal endoscopic approach to remove these lesions in a lower medial location of the orbit, a location similar to the lesion reported in this study. The authors state that external surgical approaches, such as external orbitotomy, can be performed with or without osteotomy and guarantee satisfactory results. However, an alternative approach that is still scarce in the literature—needing more results based on scientific evidence—would be the transnasal endoscope, considered a viable and safe option if the medial rectus muscle is effectively managed for the exposure of the intraconal space. 9 10

Nonsurgical methods are recommended for small, asymptomatic, and slow-growing masses. 5 Sclerotherapy is a nonsurgical treatment option indicated for complex hemangiomas, which are difficult to be resected because they are positioned deeply in the orbit. Or in cases where the predictability of damage of muscles and nerves is high. 7 However, surgical excision is necessary for the definitive diagnosis.

The recurrence rate is rare after surgical excision, but it has been reported in the literature, 5 as in this case, in which the patient presented a recurrence of the lesion after the first excision.

Surgical treatment of HCOs is indicated only for symptomatic patients with proptosis, diplopia, pain, and reduced visual quality. 8 The appearance of postsurgical complications is causally related to the extent to which the HCO capsule is linked to important critical structures in this region. If this separation/removal is successful, the treatment may have a good prognosis. 2 In this sense, the patient must be informed about possible complications, such as profound loss of vision, diplopia, ptosis, corneal anesthesia, pupillary abnormality, loss of accommodation, and any risks resulting from the chosen surgical procedure. 8

Based on its rate of achievement and success, lateral orbitotomy is considered the most common surgical approach, as it provides an excellent exposure of tumors located in the upper, lateral, or lower compartment of the orbit. 1 In this sense, the HCO, in the patient reported in this study, was in the lower compartment, thus employing an inferior lateral orbitotomy approach with a bone flap, leading to a complete and successful removal of the lesion. 1 7

In this perspective, it is observed in the literature the report of several cases of orbicular cavernous hemangioma between men and women, aged 20 to 60 years old, who presented similar signs and symptoms and underwent imaging tests and surgical treatment for injury removal and risk reduction. 1 2 3 4 5 6 7 8 11

Among these authors, Yan and Wang 8 report on a 54-year-old male, with no significant past medical history, who sought medical care due to the presence of proptosis in his left eye with 6 days of evolution. The patient underwent a Doppler ultrasound and CT, resulting in the identification of a well-defined round mass in the left lower-anterior orbit with a slight blood flow within the mass, which extended along the orbit floor. Thus, it was decided to perform an anterior orbitotomy under local anesthesia by using an incision in the lower eyelid skin. The patient is evolving.

Regarding the injury recurrence in the case reported here, Choudhri et al 1 report on a 35-year-old male with a significant past medical history for congenital glaucoma and an anterior left orbicular cavernous hemangioma, removed 28 years ago through a lateral orbitotomy. A supra-orbital mass was diagnosed, and it was then decided to perform a left lateral orbitotomy due to the progression of ocular abnormalities and the growth of recurrent hemangioma. About 2 years after resection of the orbital hemangioma, the patient remains stable, with no signs of recurrence of the lesion but signs of ocular motor restriction, light diplopia, and inability to close the eye remained. In this sense, the patient in this study has not yet shown signs of recurrence after the second surgery and is therefore being followed up.

## Conclusion

Orbicular cavernous hemangioma is considered a common diagnosis for adult patients with evolution of proptosis and presence of an orbital mass. The surgical approach must be individualized based on the peculiarities of each case. The treatment will be successful due to the degree of fusion between the lesion capsule and important structures, such as motor and sensory nerves and muscles. If the lesion is not completely removed, the risk of recurrence is increased, and the patient must have its evolution monitored. In the present case report, the patient had a successful diagnosis and therapeutic conduct, remaining now in the evolution and follow-up scenario.
